# Placental chorioangioma and pregnancy outcome: a ten-year retrospective study in a tertiary referral centre

**DOI:** 10.1186/s12884-023-05719-x

**Published:** 2023-05-25

**Authors:** Hongwei Ma, Ziling Liu, Jie Ruan

**Affiliations:** Department of Obstetrics and Gynecology, Key Laboratory of Birth Defects and Related Diseases in Women and Children of Ministry of Education, West China Second Hospital, Sichuan University, Chengdu, 610041 Sichuan China

**Keywords:** Placental chorioangioma, Large placental chorioangioma, Perinatal complication, Preterm birth, Long-term prognosis

## Abstract

**Background:**

Placental chorioangioma is a rare disorder in pregnancy. We retrospectively reviewed the perinatal complications and long-term outcomes in pregnancies with placental chorioangioma and evaluated the factors affecting disease prognosis.

**Methods:**

We reviewed pregnant women who delivered at our hospital in the past decade and whose diagnosis of placental chorioangioma was confirmed by pathological diagnosis. Information on maternal demographics, prenatal sonographic findings and perinatal outcomes was obtained by reviewing the medical records. In the latter part of the study, follow-up of children was conducted by phone interview.

**Results:**

In the 10 years from August 2008 to December 2018, 175 cases(0.17%) were identified as placental chorioangioma histologically and 44(0.04%) of them were large chorioangiomas. Nearly one-third of cases with large chorioangiomas were associated with severe maternal and fetal complications or required prenatal intervention. Although one-fifth of fetuses/newborns complicated with large chorioangiomas were lost perinatally, the long-term prognosis for surviving fetuses was generally good. Further statistical analysis revealed that tumor size and location affect prognosis.

**Conclusion:**

Placental chorioangioma may cause an unfavorable perinatal outcome. Regular ultrasound monitoring can provide the tumor characteristics which can be referred to for predicting the tendency of those complications and indicate when intervention may be necessary. It is not clear which factors lead to complications with fetal damage as the main manifestation or polyhydramnios as the main manifestation.

## Background

Placental chorioangioma is the most common nontrophoblastic tumor of the placenta, occurring in 0.41-1.4% of examined placentas by microscopy [1]. It represents malformed differentiation of local tissue with excessive proliferation of blood vessels in chorionic villi. In histological terms, they consist of small blood vessels that are embedded within the stroma of enlarged placental villi and are covered with a trophoblast layer [2]. Most of the placental chorioangiomas were small(< 4 cm in diameter), however, large placental chorioangiomas (≥ 4 cm in diameter) were less common, with an prevalence varied from 1/9000 to 1/50,000 [3].

Gray-scale sonography, color flow Doppler ultrasonography, and magnetic resonance imaging(MRI) can be used to diagnose placental chorioangioma prenatally. Ultrasound findings of chorioangioma commonly reveal a hypo -or hyperechoic well-circumscribed placental mass. Necrosis, degeneration, or calcification of the tumor may result in different ultrasonic echotexture. Application of color flow Doppler can confirm the vascular component of placental masses and differentiate chorioangiomas from other placental lesions. Placental chorioangioma appears as a heterogeneous mass on MRI, which can be used in conjunction with ultrasound findings [4].

Many pregnancies with small placental chorioangiomas were asymptomatic and do not complicate the course of the pregnancy. Large placental chorioangiomas, by contrast, tend to be associated with perinatal complications and adverse fetal outcomes, such as polyhydramnios, preterm labor, fetal anemia, fetal growth restriction (FGR), fetal cardiomegaly or fetal heart failure, fetal hydrops, maternal mirror syndrome and even fetal demise [4]. However, the long-term effects on older infants and children, as well as factors affecting disease prognosis, are not really clear. In this 10-year retrospective study, perinatal complications as well as long-term outcomes of large chorioangiomas will be discussed for a better understanding of this disease.

## Methods

### Study population

A retrospective study was designed from 102,399 pregnant women who were admitted to the Department of Obstetrics, West China Second University Hospital from Aug. 2008 to Dec. 2018. Specimens of 8704 placentas with 10% formalin fixation and their chips with paraffin embedding were cut into 4–5 μm thick tissue slices and stained with Hematoxylin-Eosin staining. By searching pathological results “= placental chorioangioma ” in the hospital electronic patient records database, 175 cases were identified histologically and 44 of them were large chorioangiomas. The medical records, as well as a series of color Doppler ultrasound examinations, of all the included cases, were reviewed for further analysis.

### Data collection

The following data were retrieved from the pathological results and pathological pictures: the size of the tumor, and the distance from the tumor margin to the umbilical cord insertion point. The following data were retrieved from the series of color Doppler ultrasound examinations: the gestational age (GA) at first diagnosis, the size of the tumor, the blood supply of the tumor, amniotic fluid amount, middle cerebral artery peak systolic velocity(MCA-PSV), umbilical artery(UA) Doppler, presence of fetal effusions, heart size, fetal cardiothoracic ratio, cardiovascular profile score (CVPS). Maternal demographics and perinatal outcome were retrieved from the medical records: maternal age, gravidity, parity, complication, GA at delivery, mode of delivery, Apgar score of the newborn, birthweight, and the volume of postpartum hemorrhage.

### Maternal and perinatal outcomes

Primary maternal complications included polyhydramnios (defined as amniotic fluid depth(AFD) > 8 cm or amniotic fluid index(AFI) > 25 cm on ultrasound), preterm premature rupture of membrane (PPROM, defined as spontaneous rupture of membrane before the onset of labor prior to 37 weeks gestation), mirror syndrome (defined as maternal edema associated to fetal hydrops), hypertensive disorders in pregnancy and postpartum hemorrhage (defined as the amount of blood loss exceed 500mL after delivery). Primary perinatal complications included preterm birth(PTB), fetal anemia (highly suspected when MCA-PSV value ≥ 1.5 times the median), fetal growth restriction or small for gestational age(SGA) at birth, fetal cardiomegaly or fetal heart failure (CVPS < 7), fetal hydrops (defined as abnormal accumulation of fluid in two or more fetal compartments-the presence of ascites, pleural effusion, pericardial effusion, or generalized skin edema), fetal distress(defined as the presence of any one of: negative normal stress test, positive oxytocin challenge test, biophysical profile score ≤ 4, Apgar score ≤ 7, or meconium-stained amniotic fluid), intrauterine death (IUD) or neonatal death (NND).

At the end of 2020, phone interviews were conducted with 36 cases (43 live newborns) of large chorioangiomas. Information on mental development and psychomotor development, as well as developmental complications, were collected based on the parents` description. Three patients were lost to follow-up.

## Results

The prevalence of placental chorioangioma in our centre is 0.17% (175/102,399 ). The result may have been underestimated because most of the microtumors were diagnosed by accident after biopsy, by inference, similar lesions may also exist in placentas not sent for pathological examination. However, large chorioangioma was difficult to miss in the general examination, and the prevalence of large chorioangioma (0.04%, 44/102,399) seems reliable.

**Fig. 1:**
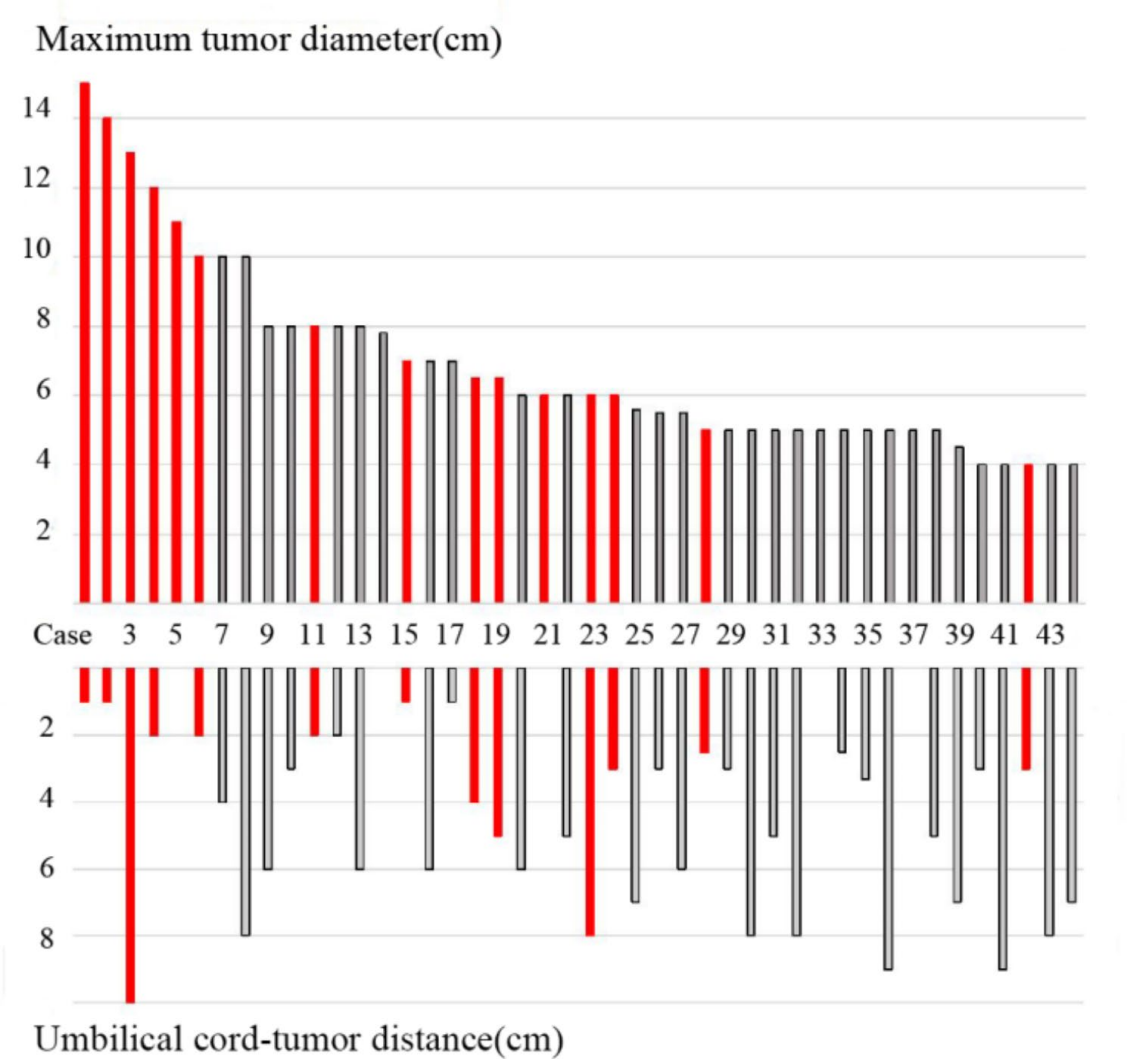
Features of 44 cases with large placental chorioangioma. The bar chart in the top half represents the maximum tumor diameter and the bar chart in the bottom half represents the distance between the tumor edge and the umbilical cord insertion point. Cases in red suffered from severe maternal and fetal complications or require prenatal intervention, while cases in black went through normal pregnancy processes

Most of placental chorioangiomas (102/177) were microscopically small (< 1 cm) and discovered only during histological inspection of the placenta, while in 29 cases were 1-3.5 cm in size, and in 44 cases the maximum diameter of tumors were larger than 4 cm(see in Fig. 1, number these 44 cases from *case 1* to *case 44* in descending order of tumor size). The largest tumor (*case 1*) was 15 × 12 × 12 cm.

Details of 44 large placental chorioangiomas were analyzed. Most of the tumors were diagnosed before delivery, however, seven cases failed to be detected by serial ultrasound examinations and were only diagnosed postpartum with the maximum diameter of the tumor less than 6 cm. The median GA at the first presentation by ultrasound in the other 37 cases was 28 + 5 ( range, 17 + 6 to 37 + 6 ) weeks.

**Fig. 2:**
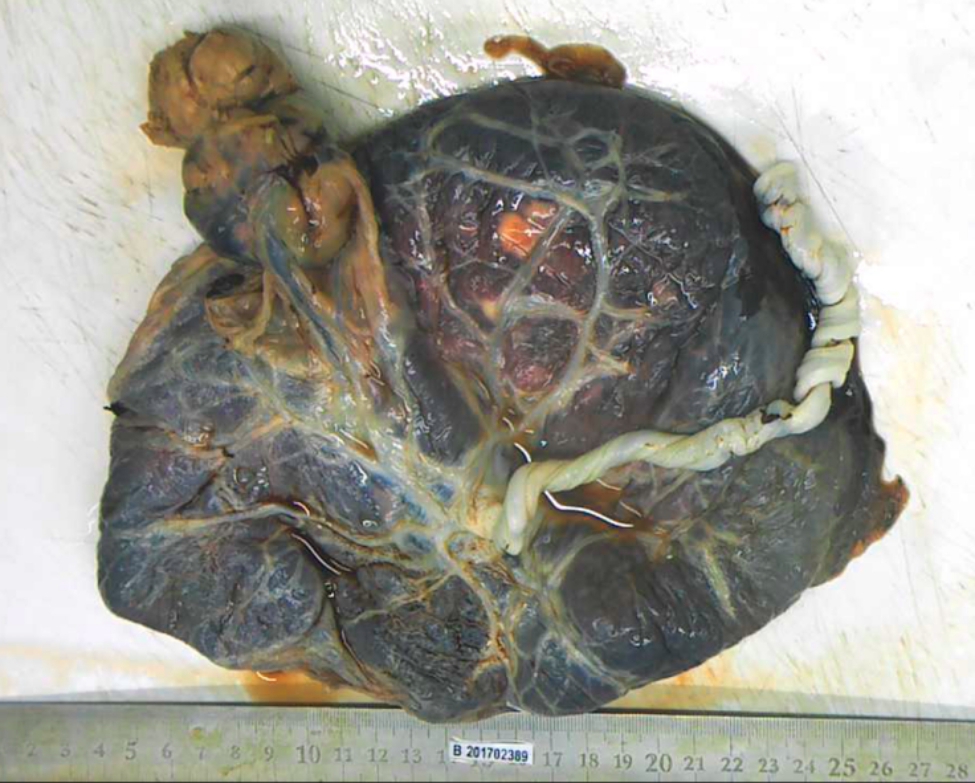
Pedunculated placental chorioangiomas in *case 23*. Shot on March 2017

Multifocal placental lesions were found in four cases of large chorioangiomas. 59.1%(26/44) of the large chorioangiomas were paracentral located in the placenta parenchyma; 38.6%(17/44) were localized on the outer edge. In *case 23*, the tumor was pedunculated, that is, protruding into the amniotic cavity and connected to the placenta by a slender vascular pedicle(Fig. 2). And in *case 21*, The umbilical cord passes through the tumor and insert in the placenta.

84.1%(37/44) occurred in singletons, and 15.9% (7/44) occurred in twin pregnancies. The 44 patients were between the age of 20 and 40, with a median age of 29.6 y/o. Most of the patients (36/44, 81.8%) were under 35 years old, while eight patients were in the 35 to 40 age bracket. Only 6 patients had previous childbirth history.

Severe maternal and fetal complications occurred in 15 cases. Details were shown in Table 1. Polyhydramnios occurred in 12 cases, with a max AFI of 41.3 cm, among which 2 cases required amnioreduction, and 1 case was given oral indomethacin. Five cases had preterm premature rupture of membranes and were treated by tocolytic agents. One patient(*case 21*) had a high fever and the possibility of intrauterine infection. Only one case(*case 14*) of preeclampsia was found.

9 fetal/neonatal losses of large placental chorioangiomas were observed in our retrospective study, including 3 lethal induction of labor, 5 IUDs, and 1 NNDs. In *case 1*, the patient was referred at 32 + 3 weeks because of threatened premature labor and maternal mirror syndrome, which was characterized as hypoxemia (lowest oxygen saturation of 72%), cyanosis, edema, abdominal distention, proteinuria, hypoproteinemia(lowest serum albumin 20 g/l), moist rales of both lungs, and patchy opacity of lower lungs on X-ray scan. At referral, the fetus had nonimmune hydrops(pericardial, pleural effusion and ascites, scalp edema), thickened placenta(7.6 cm), and polyhydramnios. Given the severe maternal situation, none of the tocolytic agents was used, and after the demand of parents, craniotomy during labor proceeded because of the possible poor prognosis of the newborn. 24 h after the delivery, the maternal symptoms were obviously relieved. In *case 4*, lethal induction of labor after the demand of parents was also operated on account of the fetal cardiomegaly, fetal pericardia effusion, and polyhydramnios. In *case 28*, the fetus was the only one who underwent amniotic fluid culture for whole-exome sequencing prenatal diagnosis in 44 cases of large chorioangiomas, for concomitant complex congenital heart disease(aortic coarctation and hypertrophic cardiomyopathy). Lethal induction of labor was also demanded because of RAF-1 gene mutation to indicate the possibility of Leopard spot syndrome and Noonan syndrome. Because of central placenta praevia with placental implantation, the bleeding during delivery was about 1330 ml, and this was the only case of postpartum hemorrhage in the large placental chorioangioma group. In *case 5*, the patient was admitted for an increased MCA-PSV value of 2.13MoM. Chorioangioma radiofrequency ablation was planned. Sadly, fetal demise happened before the scheduled surgery. In *case 6*, despite the timely use of ritodrine hydrochloride, premature breech delivery still occurred in 10 h after PPROM, which was associated with an intrapartum stillbirth. Similarly, in *case 15*, stillbirth during breech delivery was also recorded despite the use of ritodrine hydrochloride and amnioreduction against the AFD of 15.2 cm. In *case 24* and *case 42*, fetal demise happened in the late third trimester. Chorioangiomas failed to be detected by ultrasound in the two cases so ultrasonic monitoring was not so frequent. Before fetal demise, ultrasound was not taken for more than 4 weeks in *case 24* and more than 6 weeks in *case 42*, which makes the analysis of maternal and fetal complications difficult.

The median GA at delivery for the other 36 cases with alive newborns was 37 (range, 28 + 4 to 40 + 5 ) weeks. Preterm deliveries before 37 weeks, 34 weeks, and 32 weeks were 30.6%(11 cases), 13.9%(5 cases), and 11.1%(4 cases), respectively. Neonatal intensive care unit (NICU) Admission of newborns was 23.3%(10 newborns in 7 cases, including 3 twin pregnancies).In *case 21*, the baby was delivered at 29 + 3 week`s gestation, suffering from neonate respiratory distress syndrome, neonatal shock, neonatal sepsis, severe neonatal anemia(hemoglobin 85 g/l), metabolic acidosis and dysfunction of coagulation that led to the newborn death. Other severe neonatal complications included neonatal intracranial hemorrhage(NIH), hydrocephalus, bronchopulmonary dysplasia(BPD), and renal function damage.

**Table 1:** Severe perinatal complications in cases of large placental chorioangiomas

Case		GA at delivery/weeks	Maternal complications	Fetal complications	Pregnancy/Children Outcome
1	Singleton	32^+ 3^	Mirror syndrome, Polyhydramnios	Fetal hydrops	Iatrogenic lethal induction of labor
2	Twin pregnancy	34^+ 3^	-	Fetal cardiomegaly, Fetal hydrops, Fetal distress, PTB	NICU admission (7 days/28 days), Normal development
3	Singleton	33^+ 6^	PPROM	Fetal cardiomegaly (transplacental digoxin intervention), Fetal anemia, PTB	NICU admission (13 days), NIH (grade III), Normal development
4	Singleton	25^+ 4^	Polyhydramnios	Fetal cardiomegaly, Fetal hydrops	Patient asked for lethal induction of labor
5	Singleton	25	-	Fetal cardiomegaly, Fetal hydrops, Fetal anemia, IUD	
6	Singleton	28^+ 4^	Polyhydramnios, PPROM	PTB	Stillbirth during labor
11	Singleton	28^+ 4^	Polyhydramnios, PPROM	Fetal hydrops, Fetal anemia, PTB	NICU admission (61 days), NIH (grade II) and secondary hydrocephalus, Poor performance of left-hand fine motor functions
15	Singleton	27^+ 2^	Polyhydramnios (amnioreduction intervention),	PTB	Stillbirth during labor
18	Singleton	29^+ 4^	Polyhydramnios	PTB	NICU admission (46 days), NIH (grade IV), Claudication of the left lower extremity, BPD, infantile cutaneous hemangioma, Transient renal function damage
19	Twin pregnancy	31^+ 5^	Polyhydramnios, PPROM, Placental abruption	PTB	NICU admission (36 days/32 days), Normal development
21	Singleton	29^+ 3^	Polyhydramnios, PPROM and chorioamnionitis	Fetal anemia, PTB	NICU admission (2 days), Neonatal shock, NND
23	Singleton	38^+ 2^	Polyhydramnios (amnioreduction and oral indomethacin intervention)	-	Normal development
24	Singleton	38^+ 1^	-	IUD	
28	Singleton	33^+ 3^	Polyhydramnios, PPROM, PPH	Fetal hydrops, fetal abnormality	Iatrogenic lethal induction of labor
42	Singleton	37	Polyhydramnios	IUD	

* GA: gestational age; PTB: preterm birth; NICU: neonatal intensive care unit; PPROM: preterm premature rupture of membrane; NIH: neonatal intracranial hemorrhage; IUD: intrauterine death; NND: neonatal death; BPD: bronchopulmonary dysplasia; PPH: postpartum hemorrhage.

Nearly all alive babies had good outcomes. The follow-up period ranged from 1 to 12 years, 3 cases(*case 8*, case *10*, and *case 13*) failed to follow up. The most common newborn complication was neonatal benign hemangiomatosis, with five cases affected but different prognoses. *Case 33* developed a single 4 cm-in-diameter hemangioma on the head skin, which had neither expanded nor shrunk at the follow-up time when the child was 15 months old. Likewise in *case 22*, hemangioma on the bottom of newborn feet remained when the child grew to 18 months old. By contrast, In *case 35*, a coin-size hemangioma on the baby`s head skin gradually disappeared within 1 year. Similar trend was observable in *case 18*, hemangioma on the right thigh vanished quickly after discharge from NICU. And in *case 34*, most of the 16 scattered hemangiomas disappeared within 1 year, finally leaving a 5 mm-in-diameter hemangioma on the child`s left palmar skin, and the size of the primary lesion was substantially decreased. No intrahepatic lesions were observed in all newborns in our research. Fetuses of *case 2* and *case 3* were complicated with cardiomegaly. Termination of pregnancy was decided for the former based on the negative normal stress test and larger gestational weeks. C*ase 3*, who suffer from fetal cardiomegaly since the early stage of 3rd trimester, received maternal transplacental administration of digoxin at 32 + 3 week`s gestation. Echocardiography of newborns of the 2 cases manifested cardiac cavity with normal size before discharge from NICU. None of the children complained of cardiac symptoms at 2–3 years follow-up evaluation. All 3 premature infants with NIH received rehabilitation training. The baby of *case 3* grew and developed well at 2 years follow-up. In *case 11*, hydrocephalus secondary to gradeII NIH and infant fine motor delay was observed in the premature baby. In *case 18* with grade IV NIH, who had a regular follow-up in the children’s rehabilitation department in our hospital, normal language development and slight claudication of left lower extremity at 2 years follow-up was observed. The motor development of the upper limb, right lower limb, and fingers was good. Delay of gross motor function development and short stature occurred in *case 29* with normal pregnancy process.

To further explore whether tumor size and location affects prognosis, data were compared between the 15 cases with severe perinatal complications and the other 29 cases with relatively good outcomes. Because the data disagreed with normal distribution and homogeneity of variance, the statistical method of non-parametric rank sum test(wilcoxon Man-whttey test) was adopted with the application of the statistical package of SPSS 26.0 to carry out data processing analysis. As shown in Tables 2 and 3 below, the maximum tumor diameters and the distances between the tumor edge and the umbilical cord insertion point had statistical differences between the two groups.

**Table 2:** Comparison of maximum tumor diameter in 2 groups

Maximum tumor diameter	M(*P*_25_, *P*_75_)	wilcoxon Man-whttey test	
		Z	*P*
15 cases with severe maternal or fetal complications	(6,12)	−2.764	0.006
29 cases with relatively good outcomes	(5,7.4)		

**Table 3:** Comparison of umbilical cord–tumor distance in 2 groups

Umbilical cord–tumor distance	M(*P*_25_, *P*_75_)	wilcoxon Man-whttey test	
		Z	*P*
15 cases with severe maternal or fetal complications	(1,4)	−2.239	0.025
29 cases with relatively good outcomes	(3,7)		

A retrospective review of 102 cases of small chorioangioma (< 1 cm) and 29 cases of medium chorioangiomas (1-3.5 cm) was also performed. 2 fetal/neonatal losses were observed in the small tumor group, due to twin-twin transfusion syndrome and early onset severe preeclampsia. One fetal demise occurred at 38 + 3 gestational weeks with a tumor size of 2 cm, and umbilical vein thrombosis was confirmed by pathological examination. The median GA at delivery was 35 weeks in the small chorioangioma group and 36 weeks in the medium tumor group, respectively.

## Discussion

According to previous literature, large chorioangioma is associated with a high rate and variety of perinatal complications. Zanardini et al. surveyed 19 cases of large placental chorioangioma, in which the natural history, intrauterine treatment, and outcome of pregnancy were evaluated. Two-thirds developed complications that required either elective delivery for fetal growth restriction or intervention for tumor-related effects [3]. Dong et al. examined 56 cases of placental chorioangioma (≥ 2.2 cm in diameter), the incidence of polyhydramnios, preeclampsia, fetal distress, preterm birth, fetal loss or induced abortion was 16.1%, 8.9%, 8.9%, 23.2%, and 10.7%, respectively [5]. Wou et al. described 23 cases diagnosed with chorioangioma, of these, 14 cases with a chorioangioma < 2 cm in size. The rate of NICU admission was significantly increased for the chorioangioma group(26.1% vs. 7.3% for the control group) [6]. In Liu et al.`s study, eleven of the 12 cases with a tumor diameter larger than 4 cm were associated with complications, whereas only one of the 4 cases with small chorioangioma was complicated [7]. Similarly, in our study, nearly one-third of large placental chorioangiomas developed severe maternal or fetal complications, and one-fifth were related to fetus loss.

But we also found, in previous literature and our study, partial cases had a benign process. What are the key factors in the prognosis? In a systematic review and meta-analysis including twenty-eight studies and 161 pregnancies, the size of the mass and presence of fetal hydrops were found to be the main determinants of perinatal outcome in pregnancies complicated by chorioangioma [8]. In our study, the maximum tumor diameters were verified as one of the factors by statistics. The mechanism could be due to the formation of a vascular communication between the fetal circulation and the rapidly grown tumor, by means of a “stealing” phenomenon, which short circuits blood from a high to a low-resistance vascular bed. The larger the tumor size, the more the shunt amount, finally leading to fetal anemia, heart failure, and fetal hydrops [9]. On the other hand, the large tumor can act as a physiological and functional dead space; this may lead to uteroplacental insufficiency with subsequent chronic hypoxia, fetal distress, growth restriction, and fetal demise [3]. Other possible mechanisms included the placental transudate hypothesis. Excessive transudation of fluid from the large tumor surface leads to polyhydramnios and related complications [3].

Based on the concept of blood shunt, some researchers hypothesized that the vascularity of the tumor may be another independent factor of fetal complications regardless of the tumor size. Avascular chorioangiomas with less blood supply may have little effect on fetal blood circulation [7]. It suggests color flow and power-Doppler flow imaging may give valuable information on the prognostic evaluation of large chorioangioma. However, given the lack of grading evaluation standard of the ultrasonic intensity of chorioangioma vessels, this seems unlikely to be operated by routine ultrasound. Three-dimensional color Doppler acquisition and placental MRI has recently been reported as adjunct to placental imaging, which can offer more information about the tumor and its vascular distribution [10, 11].

Since the overall blood supply of the tumor is not easy to assess, some indirectly evaluating indicator was assumed to reflect chorioangioma vascularity. Increasing umbilical cord-tumor distance lead to less blood flow and smaller vessel diameter, so the fetus will be less affected by the chorioangioma. Sepulveda et al. reported 3 cases of large placental chorioangiomas managed with endoscopic laser coagulation of the feeding vessels; in two cases with poor pregnancy outcomes, the chorioangioma was close to the umbilical cord insertion and the feeding vessels were voluminous [12]. Similarly, we noticed statistical differences in the umbilical cord-tumor distance between the 2 groups with adverse outcomes and good outcomes. This result was consistent with the observation results of Liu et al. [7].

However, it can still be found that some cases may not follow the above rules. As in *case 23*, although the size of the tumor was not particularly large and the tumor was far enough away from the umbilical cord, complications still occurred. It is speculated that the pedunculated tumor may have a large contact area with the amniotic cavity, which caused more transudation of fluid and eventually excessive amniotic fluid.

Complications can manifest in various forms and, in general, fall into two categories, one with fetal intrauterine damage as the main manifestation, including fetal cardiomegaly, hydrops, anemia, distress, etc. In the other group of patients, no intrauterine fetal damage is found, but only polyhydramnios, PPROM, and PTB are the main manifestations, and the effects in children often come from the PTB itself. It is unclear which factors determine the exact form of complications and more research are needed.

Treatment of complications depends on gestational age and mater-fetal symptoms. Termination of pregnancy should be considered for worsening maternal condition, or late-onset complications with viable fetuses. If severe complications occur before the fetus is viable, prenatal intervention should be carried out to extend the gestation, which is classified as supportive and definitive treatment. The choice of supportive treatment is based on the specific type of complication, such as intrauterine transfusions for anemic fetuses and hydropic fetuses, amnioreduction and oral indomethacin for polyhydramnios with respiratory embarrassment and/or preterm labor, transplacental digoxin intervention for fetal cardiomegaly. Definitive treatment refers to the treatments to block arteriovenous shunting including alcohol injection, microcoil embolization, endoscopic laser coagulation, radiofrequency ablation, and interstitial laser therapy. In this study, intrauterine supportive treatment was performed in a limited number of cases with mostly satisfactory outcomes. Unfortunately, no cases in this study underwent definitive treatment. One patient was scheduled for radiofrequency ablation for fetal anemia. However, the fetus died preoperatively due to the rapid progression of the disease. To date, there is still no consensus on how to choose the appropriate treatment for a given complication. More research is needed on whether to choose supportive or definitive treatment, and whether to choose a single treatment modality or combined therapy.

## Conclusion

To our knowledge, this study is one of the research with the largest number of cases and longest period of follow-up of large placental chorioangioma. Using this advantage, we can better understand the full spectrum of the illness and its complications. Nearly one-third with large placental chorioangioma suffer from severe perinatal complications. Regular ultrasound monitoring can provide the tumor characteristics which can be referred to for predicting the tendency of those complications and indicate when intervention may be necessary.

## Data Availability

The datasets used and/or analyzed during the current study are available from the corresponding author on reasonable request.

## Abbreviations

FGR: fetal growth restriction
GA: gestational age
MCA-PSV: middle cerebral artery peak systolic velocity
UA: umbilical artery
CVPS: cardiovascular profile score
AFD: amniotic fluid depth
AFI: amniotic fluid index
PTB: preterm birth
SGA: small for gestational age
IUD: intrauterine death
NND: neonatal death
NICU: neonatal intensive care unit
NIH: neonatal intracranial hemorrhage
BPD: bronchopulmonary dysplasia
PPROM: preterm premature rupture of membrane
PPH: postpartum hemorrhage.
